# Scanning mutagenesis of the I-II loop of the Ca_v_2.2 calcium channel identifies residues Arginine 376 and Valine 416 as molecular determinants of voltage dependent G protein inhibition

**DOI:** 10.1186/1756-6606-3-6

**Published:** 2010-02-03

**Authors:** Hugo W Tedford, Alexandra E Kisilevsky, Lucienne B Vieira, Diego Varela, Lina Chen, Gerald W Zamponi

**Affiliations:** 1 Department of Physiology and Pharmacology, Hotchkiss Brain Institute, University of Calgary, Canada

## Abstract

Direct interaction with the β subunit of the heterotrimeric G protein complex causes voltage-dependent inhibition of N-type calcium channels. To further characterize the molecular determinants of this interaction, we performed scanning mutagenesis of residues 372-387 and 410-428 of the N-type channel α_1 _subunit, in which individual residues were replaced by either alanine or cysteine. We coexpressed wild type Gβ_1_γ_2 _subunits with either wild type or point mutant N-type calcium channels, and voltage-dependent, G protein-mediated inhibition of the channels (VDI) was assessed using patch clamp recordings. The resulting data indicate that Arg^376 ^and Val^416 ^of the α_1 _subunit, residues which are surface-exposed in the presence of the calcium channel β subunit, contribute significantly to the functional inhibition by Gβ_1_. To further characterize the roles of Arg^376 ^and Val^416 ^in this interaction, we performed secondary mutagenesis of these residues, coexpressing the resulting mutants with wild type Gβ_1_γ_2 _subunits and with several isoforms of the auxiliary β subunit of the N-type channel, again assessing VDI using patch clamp recordings. The results confirm the importance of Arg^376 ^for G protein-mediated inhibition and show that a single amino acid substitution to phenylalanine drastically alters the abilities of auxiliary calcium channel subunits to regulate G protein inhibition of the channel.

## Background

The mammalian nervous system expresses nine different genes that encode different types of voltage-gated calcium channel (VGCC) α_1 _subunits which interact with auxiliary subunits and form classes of VGCCs that are distinct in structure, pharmacology, and physiology [1]. VGCCs containing the α_1A _and α_1B _subunits (P/Q- and N-type channels respectively) are distinguished from other types by their localization to pre-synaptic nerve terminals, where they mediate calcium influx which contributes to evoked neurotransmitter release and overall synaptic function [2-4].

Inhibition of P/Q- and N-type channels resulting from activation of G-protein coupled receptors (GPCRs)--a crucial mode of regulation, notably illustrated in the relief of pain sensations in response to opioid drugs [5]--has been studied for over 25 years [6-16]. This mode of regulation is complex and comprises multiple pathways that include direct and indirect actions of G proteins on the channel [17]. During membrane delimited G protein inhibition, GPCR activation releases Gβγ heterodimers which then bind directly to the α_1 _subunits of P/Q- and N-type channels, and this interaction stabilizes closed channel conformations and culminates in channel inhibition [18,19].

A recent study suggests that interaction of Gβγ with N-type channels can slow the kinetics of channel transition to inactivated states [20]. However, most studies of the direct Gβγ-presynaptic channel interaction have investigated the slowing of transition to activated channel states, and have found this mode of inhibition to be more favored at hyperpolarized potentials, thus allowing for activity dependent dis-inhibition [21-24]. Hence, the term "voltage-dependent inhibition" (VDI) has been used to describe two experimental hallmarks of this Gβγ-mediated regulation: slowing of presynaptic channel activation, and relief of channel inhibition by a strong, depolarizing pre-pulse.

Gβγ-mediated VDI depends on a complex set of structural determinants that contribute to direct interaction between Gβγ and the presynaptic calcium channel. As such, the extent of VDI varies with the isoforms of the channel subunits and the G protein subunits in question [17]. Structure-activity relationship studies of the interaction have revealed roles for three cytosolic regions of the α_1 _subunit: the N-terminus, the I-II linker domain, and the C-terminus [25-30]. While the C-terminal region of the channel is thought to play a supporting role as an enhancer of overall Gβγ-channel binding affinity [31], both the N-terminus and the I-II linker contribute directly to the inhibitory interaction with Gβγ. Furthermore, contact between the N-terminus and the I-II linker is demonstrated to be necessary for Gβγ-mediated VDI [25]. Efforts to resolve functionally important Gβγ-channel binding interactions have also revealed the direct involvement of two nearby sections of the I-II linker: amino acid residues 372-389 and 410-428 [27,30]. The first of these sections partially overlaps with the α_1 _subunit alpha interaction domain (AID) and contains residues known to bind the calcium channel β subunit, presumably in a manner that precludes many of them from interaction with Gβγ [32]. However, the contribution of the other I-II linker residues in question--to direct Gβγ-binding and hence to channel inhibition--has remained unclear.

Here we aimed to further resolve the molecular determinants of Gβγ-mediated channel inhibition by testing the functional contribution of individual residues in the two above-mentioned sections of the α_1B _I-II linker. Using a combination of alanine/cysteine scanning mutagenesis and whole-cell electrophysiological recordings from tsA-201 cells, we identify two residues of the I-II linker, Arg^376 ^and Val^416^, as key determinants of Gβγ-mediated, voltage-dependent modulation of N-type channels.

## Methods

### cDNAs

Wild type (WT) rat calcium channel subunit cDNAs encoding Ca_v_2.2 (α_1B_), Ca_v_β_1b_, Cavβ_2a_, Ca_vβ3_, and Ca_vβ4_, and α_2-_δ_1 _subunits were generously donated by Dr.Terry Snutch (University of British Columbia, Vancouver, BC). The construction of cDNAs encoding WT human Gβ_1 _and Gγ_2 _subunits have been described previously [33].

### Ca_v_2.2 α_1B _mutants

cDNAs encoding single-residue Ca_v_2.2 α_1B _mutants were constructed by overlap extension PCR [34], using WT α_1B _as the DNA template and Pfu turbo DNA polymerase (Stratagene) according to manufacturer's instructions. AarI and BsiWI restriction sites, found in the native sequence at locations flanking the mutagenized I-II loop-encoding sequence, were included in the 5' ends of the non-mutagenic flanking primers. After mutagenizing and overlap extension reactions, mutant α_1B _cDNA fragments were digested with AarI and BsiWI, and then sub-cloned into the (AarI-, BsiWI-digested) α_1B _mammalian expression vector, pCMV30-14G. Codons for 27 residues within amino acid sequence regions 372-389 and 410-428 were individually substituted to alanine, and three naturally occurring alanine codons were substituted to cysteine. These 30 mutations included: F372A, L373A, K374A, L375A, R376A, R377A, Q378A, Q379A, E382A, R383A, N386A, K410A, S411A, P412A, L413A, D414A, A415C, V416A, L417A, K418A, R419A, A420C, A421C, T422A, K423A, K424A, S425A, R426A, N427A, and D428A. cDNAs encoding four additional point mutations, R376E, R376F, V416E, and V416K, and a double alanine mutation, (both R376A and V416A), were also constructed using overlap/extension PCR as described above.

### Tissue Culture and Transient Transfection

Human embryonic kidney tsA-201 cells were grown and transiently transfected using the calcium phosphate method as described previously (32). Transfection solutions for individual culture dishes contained a mixture of cDNA expression vectors, with the following quantities of each cDNA expression construct used: calcium channel α_1B _subunit, 6 μg; Ca_v_β subunit (6 μg), Ca_v_α_2_-δ_1 _subunit (6 μg), Gβ_1 _subunit, 6 μg; Gγ_2 _subunit, 6 μg; and 1 μg of a pEGFP marker vector (Clontech). Positive controls contained the WT α_1B _subunit in place of mutant α_1B_, and negative controls consisted of the WT α_1B _subunit in the absence of exogenous Gβ_1_γ_2_. 12 hr post-transfection, cells were washed 1× with PBS pH 7.4, supplemented with fresh DMEM, and allowed to recover for an additional 12 hr. To prevent overgrowth, cells were subsequently transferred to a 29°C incubator and maintained for 24-72 hr prior to voltage-clamp recording.

### Ca_v_2.2 Voltage Clamp Recordings

Glass coverslips carrying cells expressing mutant or WT Ca_v_2.2 channels were transferred to a 3.5-cm culture dish (Corning) containing external recording solution consisting of 20 mM BaCl_2_, 1 mM MgCl_2_, 10 mM HEPES, 40 mM TEA-Cl, 10 mM glucose, and 65 mM CsCl (pH 7.2 adjusted with TEA-OH). Micro-electrode patch pipettes were pulled using a Sutter P-87 micro-electrode puller or a DMZ Universal puller, and manually fire-polished using a Narishige MF-830 Micro Forge to attain a typical resistance of 4-5 MÙ. Internal pipette solution consisted of 108 mM CsMeSO_4_, 4 mM MgCl_2_, 9 mM EGTA, and 9 mM HEPES (pH 7.2 adjusted with CsOH).

Whole cell patch clamp recordings were performed in voltage-clamp mode using an Axopatch 200B amplifier (Axon Instruments) linked to a personal computer with pCLAMP version 9.0 or 9.2 software. Series resistance was compensated by 85%, leak currents were negligible, and the data were filtered at 1 kHz. Individual pEGFP-expressing cells were held at -100 mV, and currents were evoked by stepping to a test potential of +20 mV. Only cells with current amplitudes greater than 50 pA and less than 1.5 nA were used for analysis.

Voltage-dependent G protein inhibition was assessed by application of a strong, depolarizing pre-pulse (PP) to +150 mV for 50 ms, immediately prior to the test potential--during alternating sweeps of an assay. Pre-pulse relief of inhibition was quantified as the ratio of peak current amplitudes observed in paired test pulses performed with (I_+PP_) and without (I_-PP_) the prepulse (i.e., I_+PP_/I_-PP_).

### Data Analysis

All data were analyzed using Clampfit version 9.2 (Axon Instruments) and fitted in Sigmaplot 2000 (SPSS Inc.). Statistical analyses were carried out using SigmaStat 2.03 (SPSS Inc.). All sample means are reported +/- SEM. Statistically significant differences between means were assessed using student's t-test, Mann-Whitney rank sum test, or one-way ANOVA at 95% confidence level as appropriate.

## Results

**Figure 1:**
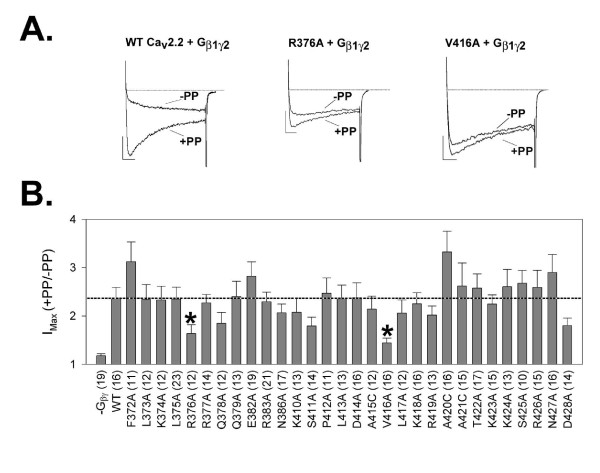
***A*: Three sets of typical *current traces *from tsA-201 cells expressing WT or mutant rat Ca_v_2.2 calcium channels and Gβ_1_γ_2_, as described in METHODS**. Each is a matched set of *current traces *from sequential test pulses, with the second test pulse preceded by a150-mV depolarizing prepulse. (Vertical and horizontal scale bars represent 15 pA and 15 ms, respectively; in each set the trace with larger current values is from the second test pulse.) *Left*: traces from a cell expressing the WT channel and Gβ_1_γ_2_. *Center*: traces from a cell coexpressing the Ca_v_2.2-R376A mutant channel and Gβ_1_γ_2_. *Right*: traces from a cell coexpressing the Ca_v_2.2-V416A mutant channel and Gβ_1_γ_2_. ***B***: Histogram summarizing the results of paired-pulse facilitation (PPF) experiments performed with all Ala/Cys point mutants of Ca_v_2.2; columns show mean PPF values with SE bars for each condition. Respective positions of mutations in the Ca_v_2.2 amino acid sequence are indicated by numbers beneath the corresponding columns (see METHODS for full description of the mutations used). Of the 30 individual amino acid residues examined in the Ca_v_2.2 I-II linker region, Ala mutations at both Arg^376 ^and Val^416 ^(*p < 0.05, t-test) result in a significant loss of Gβγ-mediated channel inhibition, as measured by the degree of pre-pulse relief following a depolarizing pre-pulse, when compared to WT control. Numbers in parentheses indicate numbers of cells tested.

Previously, two sections of the N-type channel I-II linker region, α_1B_ amino acid residues 372-389 and 410-428, were identified as functionally important binding sites for the Gβγ heterodimer [27,30]. To test the contribution of individual residues of these sections to Gβγ-mediated channel inhibition, alanine/cysteine scanning mutagensis was performed. Residues 372-389 include six amino acid residues that are predicted, on the basis of crystallographic data, to be unavailable for interaction with Gβγ, as their access is likely occluded by the calcium channel β subunit [32]. The remaining residues in this section, and in the second region (residues 410-428) were individually substituted to alanine or cysteine. The resulting mutant channels were coexpressed with human Gβ_1_γ_2_, and their respective susceptibilities to Gβγ-mediated VDI were quantified using a pre-pulse facilitation (PPF) paradigm (Figure 1).

**Figure 2:**
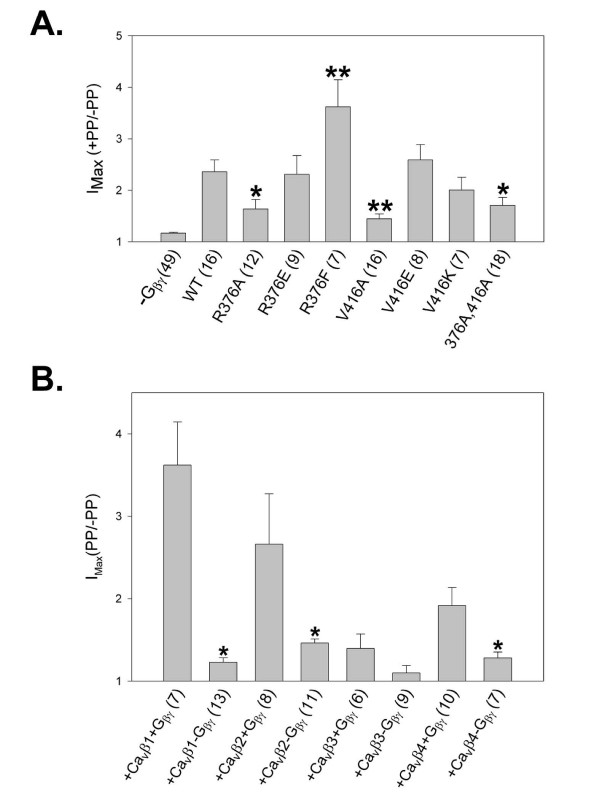
***A*: Histogram summarizing the results of PPF experiments performed with Ca_v_2.2 isoforms carrying mutations of α_1B _residues Arg^376 ^and Val^416^**. Columns show mean PPF values with SE bars for each condition. Human Gβ_1_γ_2 _was co-expressed in tsA-201 cells with the Ca_v_2.2 isoforms for each condition presented except for the negative control ("-Gβ_1_γ_2_"). Respective mutations in the Ca_v_2.2 amino acid sequence are indicated beneath the corresponding columns (see METHODS for full description of the mutations used). Of the various conditions examined only the mutations R376A, R376F, V416A, and the double mutation R376A, V416A resulted in a significant loss of Gβγ-mediated channel inhibition, as measured by the degree of pre-pulse relief following a depolarizing pre-pulse, when compared to WT control (*p < 0.05, t-test, **p < 0.05 one-way ANOVA, Dunnett's method, or Kruskal-Wallis one-way ANOVA on ranks). Numbers in parentheses indicate numbers of cells tested for the respective condition. ***B***: Histogram summarizing the results of PPF experiments using tsA-201 cells co-transfected to express α_1B _mutant R376F with Ca_v_β isoforms β_1B_, β_2_, β_3_, and β_4_, with or without heterologous human Gβ_1_γ_2 _as indicated by labels beneath columns. Columns show mean PPF values with SE bars for each condition (see METHODS for full description of the mutations used). Of the conditions examined, coexpression of Ca_v_β_1B _and Ca_v_β_2a_, and Ca_v_β_4 _resulted in statistically significant differences in mean current density for R376F channels expressed with and without heterologous Gβγ (*p < 0.05 using t-test, Mann-Whitney rank sum test, and t-test, respectively).

WT channels displayed the hallmark characteristics of Gβγ-mediated channel inhibition (Figure 1A, left), including kinetic slowing of activation and relief of inhibition by a strong depolarizing pre-pulse (PPF ratio for WT channel assays: 2.36 +/- 0.23). When examining PPF ratios obtained with mutant and WT channels, two of the 30 mutants examined, R376A and V416A, showed a significant loss of Gβγ-mediated inhibition when compared to wild type channels (PPF ratios 1.64 +/- 0.18 and 1.44 +/- 0.01, respectively; *P = 0.028 and 0.001, respectively; see Figure 1B). To test whether or not the effect of these mutations on Gβγ-mediated channel inhibition were additive, a double Ca_v_2.2 α_1B _mutant containing both the R376A and V416A substitutions was engineered. Co-expression of this double mutant with exogenous Gβ_1_γ_2_, and subsequent electrophysiological analysis using the PPF protocol, found the degree of Gβγ-mediated inhibition to be significantly less than that of WT channels, but similar to that observed in the presence of either one of the individual mutations alone (PPF ratio: 1.71 +/- 0.15, *t-test, P = 0.023) (Figure 2A).

To further characterize the roles of Arg^376 ^and Val^416^, additional single mutant α_1B _subunits, containing R376E, R376F, V416E, and V416K substitutions, were engineered and co-expressed with exogenous Gβ_1_γ_2_. Neither of the latter Val^416 ^substitutions resulted in significant changes in PPF ratio as compared to WT channels, Fig. 2A). However, the phenylalanine substitution at Arg^376 ^significantly increased the PPF ratio for channels coexpressed with Gβ1γ2 (mean values of 2.36 and 3.62 for WT and R376F, respectively, t-test p < 0.017, Fig. 2A), suggesting that this amino acid substitution actually increased Gβγ-mediated channel inhibition. These data thus further support the notion of residue 376 being an important determinant of VD G protein modulation of N-type channels.

The enhancement of Gβγ-mediated VDI by the R376F mutation, and the proximity of this mutation to critical sites of interaction between the α_1B _and β subunits of the Ca_v_2.2 channel, led us to ask whether the nature of the Ca_v_β subunit might affect this enhancement. To examine this issue, we coexpressed the α_2_-δ subunit and the R376F mutant with different isoforms of the Ca_v_β subunit in tsA-201 cells, and for each resulting population of Ca_v_2.2 channels, we tested the effect of coexpression of heterologous Gβ_1_γ_2 _on current densities and PPF ratios. As shown in Fig. 2B, little VD modulation was observed in the absence of exogenously coexpressed Gβ_1_γ_2 _irrespective of the type of calcium channel β subunit that was present. For channels containing either Ca_v_β_2a _or Ca_v_β_4_, coexpression with Gβ_1_γ_2 _induced robust VD modulation of channel activity, whose magnitude was, however, smaller than that observed with channels containing Ca_v_β_1_. Strikingly, virtually no VD modulation was evident in R376F channels containing the Ca_v_β_3 _subunit (i.e., there was no significant difference in PPF in the presence and the absence of G proteins). These data are in striking contrast to our previous findings showing that with WT Ca_v_2.2, Gβ_1_γ_2 _most strongly inhibited channels containing Ca_v_β_2a_, followed by Ca_v_β_3_, Ca_v_β_4 _and Ca_v_β_1B _[35]. Hence, a single amino acid substitution in the Ca_v_2.2 I-II linker drastically alters the Ca_v_β subunit dependence of Gβ_1_γ_2 _inhibition of the channel.

## Discussion

In this manuscript we have narrowly focused on the contribution of individual amino acids in the Ca_v_2.2 I-II linker region to voltage dependent G protein inhibition of the channel. Among thirty amino acids in the I-II linker of the Ca_v_2.2 channel, we have identified two, Arg^376 ^and Val^416^, that serve as determinants of G-protein mediated VDI of Ca_v_2.2 channels, suggesting a highly localized interaction of Gβ_1_γ_2 _with the I-II loop. The impact of single amino acid substitutions on G protein inhibition is reminiscent of our earlier findings showing that phosphorylation of a single I-II linker residue, Thr^422^, can disrupt modulation of Ca_v_2.2 channels by Gβ_1 _[36,37].

Arg^376 ^is particularly interesting because the R376F mutation drastically altered the impact of Ca_v_β subunit coexpression on the degree of VDI: whereas Gβ_1_-mediated inhibition of WT channels is reported to be strongest for channels containing Cavβ_2a_, followed by Ca_v_β_3_, Ca_v_β_4 _and Ca_v_β_1B_, respectively [35], we report here that Gβ_1_-mediated regulation of R376F channels is strongest for channels containing Ca_v_β_1B_, followed by Ca_v_β_2a_, Ca_v_β_4_, Ca_v_β_3_, respectively, with no significant voltage-dependent inhibition observed for the latter. At this point we do not know how the mutation of residue 376 to phenylalanine increases VD G protein inhibition. When coexpressed with Ca_v_β_1B _in the absence of heterologous Gβ_1_γ_2_, the R376F mutant had a half activation potential that did not differ significantly from that of the wild type channels (data not shown); moreover, at the majority of test potentials examined, the mutation yielded no significant changes in the rates of activation and inactivation (versus WT channels, data not shown), suggesting that the effects of the mutation on Gβγ modulation are not complicated by changes in biophysical properties of the channel. An increased degree of prepulse relief could occur as a result of several mechanisms. First, the mutation could destabilize the binding of Gβγ to the channel, thus resulting in more effective dissociation of Gβγ from the channel in response to membrane depolarization. This however seems unlikely, because the kinetics of the facilitated current were found to be similar for both the wild type and the mutant channels, indicating that both channels are completely dis-inhibited (and thus dissociated from Gβγ) following the application of a prepulse (data not shown). Agler and colleagues [25] reported that the N-terminus of Ca_v_2.2 is capable of interacting with the domain I-II linker, and that this interaction contributes to G protein inhibition of the channel. It is thus conceivable that the nature of residue 376 could affect G protein inhibition indirectly by virtue of altering the binding of the N-terminus to the I-II linker.

Alternatively, it is possible that residue 376 is involved in transducing Gβγ binding to alter channel gating, such that a stronger voltage dependent inhibition is observed in the mutant channel. Residue 376 is three amino acid residues just upstream of an alpha helical structure (the AID, comprising residues 379-396) that is involved in binding of the Ca_v_β subunit [38], and could potentially serve as a hinge that links G protein binding to the gating machinery of the channel. However, it has also been proposed for Ca_v_2.1 channels that I-II linker residues 357-378 are all part of a stable continuation of the alpha helical structure of the AID, and that stability and continuity of this helical structure is required for VD G protein inhibition of the channel [39]. In the latter case the R376F mutation may simply create a more stable binding pocket for Gβγ, perhaps in part by eliminating one of the eight positive charges carried by the side chains of I-II residues 357-396, which may render this section of the I-II linker less likely to move in response to a membrane depolarization event. Whatever the actual case, the proximity of Arg^376 ^to the Ca_v_β subunit interaction site also provides for a mechanism by which the nature of the Ca_v_β subunit could affect the extent of G protein inhibition that is observed.

Although alanine mutagenesis of residues 376 and 416 significantly reduced the effects of Gβγ, VDI was not completely eliminated, and the effects of the individual amino acid substitutions were not additive. This suggests that either other amino acid residues in the Ca_v_2.2 α1 subunit might help stabilize the binding of Gβγ to the channel (such as for example, residue in the N-terminus), or that the Ca_v_β subunit may contribute directly to anchoring Gβγ to the channel. The latter would be consistent with recent findings showing that the presence of the Ca_v_β subunit is required to permit VDI of Ca_v_2.1 channels [39].

Altogether, our data further implicate the domain I-II linker region as an important contributor to voltage dependent G protein modulation of N-type calcium channels. Furthermore, our results suggest that the regulation of N-type calcium channels by G proteins involves complex interactions between Gβγ, the Ca_v_2.2 α1 subunit, and the auxiliary Ca_v_β subunit, and reveal that substitution of a single amino acid residue that is conserved in all HVA calcium channels is sufficient to significantly alter the interactions among these players. Although the precise molecular mechanism by which residue 376 couples Gβγ interactions to alterations in channel function remains unknown, the observation that highly localized alteration of a single amino acid residue increased G protein inhibition of the channel may offer potential avenues to enhance the efficacy of therapeutics acting on N-type channels via GPCRs.

## Competing interests

The authors declare that they have no competing interests.

## Authors' contributions

HWT performed molecular biology, cell transfection, electrophysiology, data analysis, and proofreading. AEK performed cell transfection, most of the electrophysiology recordings, and contributed data analysis and proofreading. LC performed tissue culture and electrophysiology recordings. LBV and DL performed cell transfection, electrophysiology, and data analysis. GWZ designed and supervised the research project, and provided analysis and proofreading. All authors read and approved the final manuscript.
